# Trends and disparities in aortic dissection-related mortality among hypertensive patients in the United States (1999–2019): a nationwide analysis

**DOI:** 10.1097/XCE.0000000000000333

**Published:** 2025-05-12

**Authors:** Shehroze Tabassum, Farhan Naeem, Faraz Azhar, Aroma Naeem, Hafeez Ul Hassan Virk, Timir K Paul

**Affiliations:** a Department of Internal Medicine, King Edward Medical University, Lahore, Pakistan; b Department of Cardiology, Massachusetts General Hospital, Harvard Medical School, Boston, Massachusetts, USA; c Department of Internal Medicine, Allama Iqbal Medical College, Lahore, Pakistan; d Department of Internal Medicine, The Wright Center for Graduate Medical Education, Scranton, Pennsylvania; e Department of Cardiology, Harrington Heart & Vascular Institute, Case Western Reserve University, University Hospitals Cleveland Medical Center, Cleveland, Ohio; f Department of Cardiovascular Science, University of Tennessee Health Sciences Center at Nashville, Ascension St. Thomas Hospital, Nashville, Tennessee, USA

**Keywords:** aortic dissection, disparities, hypertension, mortality

## Abstract

Patients with hypertension (HTN) are at increased risk of aortic dissection (AoD)–related mortality. This study examined trends in AoD-related mortality among U.S. patients aged greater than or equal to 25 years with HTN. We used the Centers for Disease Control and Prevention’s Wide-ranging Online Data for Epidemiologic Research data to extract age-adjusted mortality rates (AAMRs) per 1 000 000. Trends by year, sex, age, race/ethnicity, census region, and metropolitan status were analyzed. Joinpoint regression calculated annual percent change (APC) with 95% confidence intervals. Between 1999 and 2019, there were 21 221 AoD-related deaths in patients with HTN. The AAMRs showed a rising trend from 1999 to 2006 (APC: 3.87), followed by a sharp decrease from 2006 to 2009 (APC: −18.95), and an upward trend again from 2009 to 2019 (APC: 3.52). Men (6.31), non-Hispanic Black/African American patients (9.60), West region (5.84), and metropolitan areas (4.99) had the highest AAMRs in their respective categories. These findings warrant targeted interventions and further research to address and mitigate these concerning findings.

Aortic dissection (AoD) is a life-threatening condition with significant morbidity and mortality in the USA (U.S.) [1]. Hypertension (HTN) is a well-established risk factor for AoD [2]; however, knowledge gaps remain regarding AoD-related mortality among individuals with HTN in the U.S. Evaluating the mortality burden across different regions and demographics is crucial for informing policies and addressing disparities in vulnerable populations. In this study, we aimed to assess the trends and disparities in AoD-related mortality among patients with HTN across various regions and demographics in the U.S. from 1999 to 2019.

This study utilized data from the Centers for Disease Control and Prevention’s Wide-Ranging Online Data for Epidemiologic Research (CDC WONDER) [3]. We conducted a comprehensive analysis of mortality trends related to AoD in U.S. adults aged greater than or equal to 25 with HTN from 1999 to 2019, incorporating demographic and regional variables. Using the International Classification of Diseases, 10th revision (ICD-10), we identified all fatalities where AoD (ICD-10 code: I71.0) was the underlying cause of death, with HTN (ICD-10 codes: I10-I15) as a contributing cause of death. Age-adjusted mortality rates (AAMRs) with 95% confidence intervals (CIs) were calculated per 1 million population by standardizing crude mortality rates to the 2000 U.S. standard population. The annual percent change (APC) in AAMRs, along with corresponding 95% CIs, was determined using the Joinpoint regression model to analyze mortality trends. The weighted average of the APCs was computed and presented as the average annual percent changes (AAPCs) along with the corresponding 95% CIs to summarize the mortality trend over the course of the study period. Because the CDC WONDER database provides anonymized data, institutional review board approval was not sought for this study.

From 1999 to 2019, there were a total of 21 221 AoD-related deaths among adults aged greater than or equal to 25 years having co-morbid HTN in the U.S., with an overall AAMR of 4.76/1 000 000 (95% CI: 4.70–4.83). The overall mortality trend remained stable from 1999 to 2019 (AAPC: −0.09, 95% CI: −0.89 to 0.98), with an increasing trend in AAMRs from 1999 to 2006 (APC: 3.87, 95% CI: 1.15–9.68) (*P* = 0.01), followed by a substantial decrease from 2006 to 2009 (APC: −18.95, 95% CI: −23.57 to −8.84) (*P* = 0.0004), and a noticeable increase from 2009 to 2019 (APC: 3.52, 95% CI: 1.60–6.99) (*P* = 0.001) (Graphical Abstract, Supplemental Digital Content 1, https://links.lww.com/CAEN/A70). Data on place of death showed the highest number of deaths in medical facilities (15 580, representing 73.42% of the total deaths), followed by decedents’ homes (4039, representing 19.03% of the total deaths), nursing home/long-term care (386, representing 1.82% of the total deaths), and hospice (139, representing 0.66% of the total deaths). Stratification by age group showed the highest number of deaths (10 016, representing 47% of the total deaths) among adults aged greater than or equal to 65 years with an AAMR of 11.57 (95% CI: 11.34–11.80) which was the highest when compared with adults 45–64 years (5.31, 95% CI: 5.20–5.42) and 25–44 years old (1.47, 95% CI: 1.41–1.53). Stratification by sex showed that the overall AAMR for men (6.31, 95% CI: 6.20–6.42) was higher than for women (3.30, 95% CI: 3.22–3.37). Both men and women showed a stable trend in AAMRs throughout the study period [men: (AAPC: 0.01, 95% CI: −0.80 to 1.18) (*P* = 0.810); women: (AAPC: −0.56, 95% CI: −1.49 to 0.64) (*P* = 0.356)]. Race and ethnicity stratification showed the highest AAMR among non-Hispanic Black or African Americans (9.60, 95% CI: 9.31–9.88), followed by non-Hispanic Asian or Pacific Islanders (5.32, 95% CI: 4.98–5.66), non-Hispanic Whites (4.11, 95% CI: 4.05–4.18), Hispanics or Latinos (3.71, 95% CI: 3.51–3.91), and non-Hispanic American Indian or Alaska Native population (2.88, 95% CI: 2.25–3.63). Among census regions, the West (5.84, 95% CI: 5.68–5.99) had the highest AAMR, followed by the Midwest (4.71, 95% CI: 4.58–4.85), the Northeast (4.56, 95% CI: 4.42–4.70), and the South (4.23, 95% CI: 4.13–4.33). Urbanization stratification showed that the overall AAMR in metropolitan areas (4.99, 95% CI: 4.92–5.06) was higher than in nonmetropolitan areas (3.70, 95% CI: 3.56–3.84). Across states, the AAMR was the highest in the District of Columbia (14.79, 95% CI: 12.16–17.43) and the lowest in Virginia (2.37, 95% CI: 2.08–2.66). The states in the top 90^th^ percentile were the District of Columbia, Hawaii, Vermont, New York, California, and New Mexico.

To our knowledge, this is the first study to evaluate contemporary trends in AoD-related mortality among hypertensive patients in the U.S., revealing several important findings. Though the overall mortality trend remained stable from 1999 to 2019, a significant rise in AAMRs was observed from 2009 to 2019. Men, non-Hispanic Black/African Americans, adults aged greater than or equal to 65 years, and residents of the West region, as well as those living in metropolitan areas, had the highest mortality rates.

Previous studies have shown higher AoD-related mortality among hypertensive patients [2,4]. Approximately 67.3–76.6% of patients diagnosed with AoD were reported to have HTN [5,6]. The relationship between the incidence of HTN and AoD-related mortality is significant and follows an exponentially dose-dependent pattern. The significant rise in AAMRs observed from 2009 to 2019 is a concerning trend. Several potential factors may explain this trend. First, the prevalence of uncontrolled HTN has increased after 2014 [7]. This may be linked to findings from trials like the Action to Control Cardiovascular Risk in Diabetes [8] and Secondary Prevention of Small Subcortical Strokes-Blood Pressure [9], which demonstrated no clear advantage of intensive blood pressure control. Thus, there may be a growing incidence of AoD, leading to increased AoD fatalities after 2009. Furthermore, our analysis includes deaths occurring outside medical facilities, which account for approximately 22% of total deaths throughout the study period, representing a significant portion of the study population. Another possible explanation could be the increasing life expectancy in the U.S., which has resulted in a larger population at risk [10]. Our study shows significant disparities in mortality rates based on sex, race and ethnicity, and region, highlighting the need for tailored public health strategies and the significance of social determinants of health in relation to race and ethnicity.

There are certain limitations of this study. Reliance on ICD codes and death certificates may lead to omission or misrepresentation of AoD and HTN as a cause of death. We could not conduct a subgroup analysis based on type A and type B AoD because of the unavailability of separate coding in the database. In addition, the database lacks information on disease characteristics such as laboratory values, imaging data, genetic testing, and treatment details for AoD and HTN, which may limit a thorough understanding of trends.

In conclusion, the rising AoD-related mortality among hypertensive individuals in the U.S. over the past decade (2009–2019), coupled with persistent demographic and regional disparities, underscores the need for targeted interventions and research to advance equitable health outcomes.

## Acknowledgements

The data used for this study is freely available to the public on the CDC WONDER website.

### Conflicts of interest

There are no conflicts of interest.

## Supplementary Material


