# Supplementary figures and images for: Trends and disparities in aortic dissection-related mortality among hypertensive patients in the United States (1999–2019): a nationwide analysis
Source: Cardiovasc Endocrinol Metab. 2025 May 12;14(2):e00333. doi: 10.1097/XCE.0000000000000333 (PMC12074117; doi:10.1097/XCE.0000000000000333)

Graphical Abstract


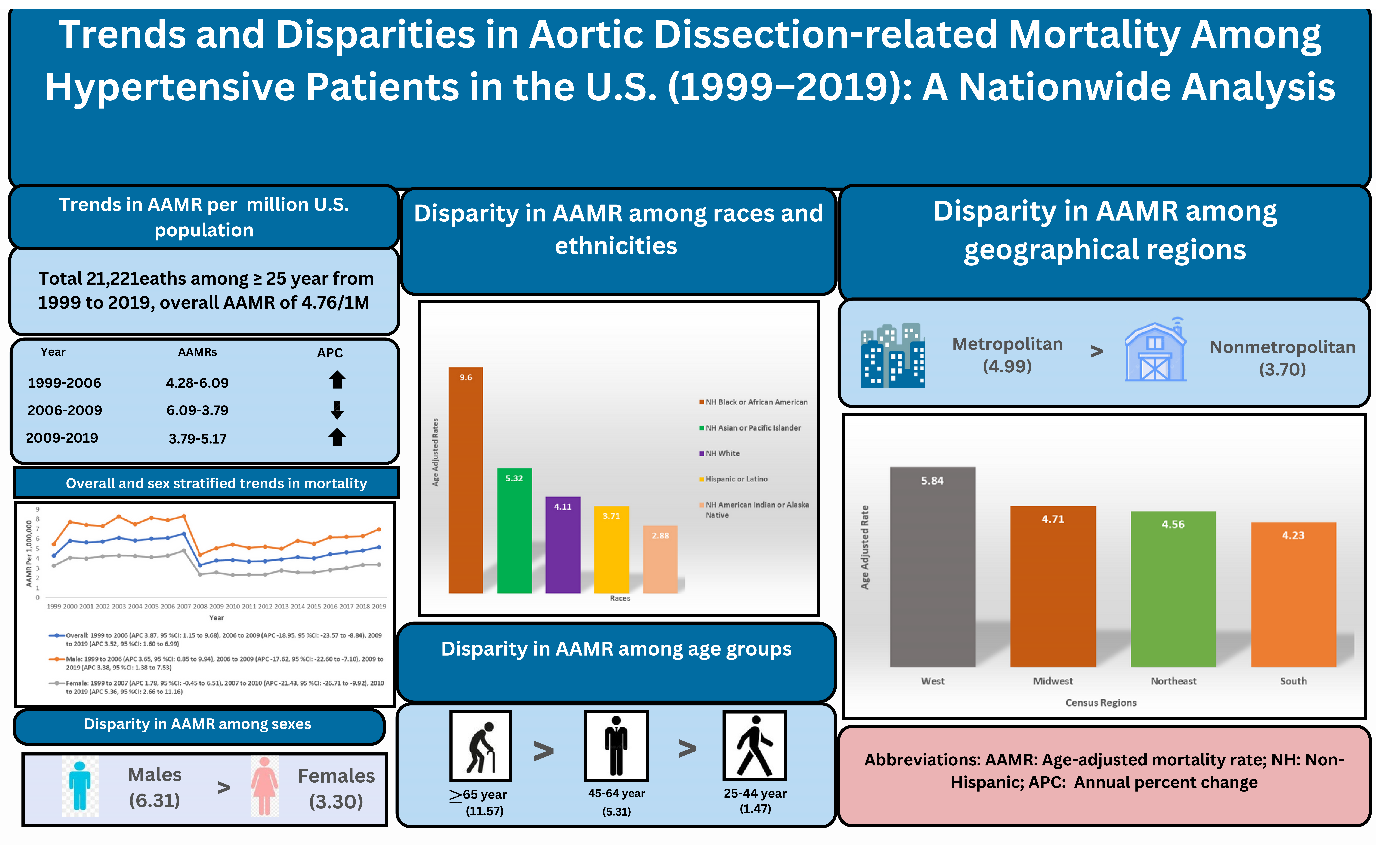

Supplement: Supplementary file 1 [file xce-14-e00333-s001.docx]
